# FTIR Spectroscopic Study of the Secondary Structure of Globular Proteins in Aqueous Protic Ionic Liquids

**DOI:** 10.3389/fchem.2019.00074

**Published:** 2019-02-13

**Authors:** Radhika Arunkumar, Calum J. Drummond, Tamar L. Greaves

**Affiliations:** School of Science, College of Science, Engineering and Health, RMIT University, Melbourne, VIC, Australia

**Keywords:** protein, ionic liquid, protic ionic liquid, lysozyme, FTIR

## Abstract

Protein misfolding is a detrimental effect which can lead to the inactivation of enzymes, aggregation, and the formation of insoluble protein fibrils called Amyloids. Consequently, it is important to understand the mechanism of protein folding, and under which conditions it can be avoided or mitigated. Ionic liquids (ILs) have previously been shown as capable of increasing or decreasing protein stability, depending on the specific IL, IL concentration and which protein. However, a greater range of IL-proteins need to be systematically explored to enable the development of structure-property relationships. In this work, the secondary structure of four proteins, lysozyme, trypsin, β-lactoglobulin and α-amylase, were studied in aqueous solutions of 10 protic ionic liquids (PILs) with 0–50 mol% PIL present. The PILs consisted of ethyl-, ethanol-, diethanol- and triethanolammonium cations paired with nitrate, formate, acetate or glycolate anions. The secondary structure was obtained using ATR-FTIR spectroscopy. It was found that lysozyme and trypsin retained its secondary structure, consistent with a native folded state, for many of the aqueous IL solutions which contained a formate or nitrate anion at the most dilute concentrations. In contrast, α-amylase and β-lactoglobulin generally had poor stability and solubility in the IL solutions. This may be due to the isoelectric point of α-amylase and β-lactoglobulin being closer to the pH of the solvents. All four proteins were insoluble in ethyl-, ethanol- and diethanolammonium acetate, though α-amylase and trypsin retained their secondary structure in up to 20 and 30 mol% of triethanolammonium acetate, respectively. It was evident that the protein stability varied substantially depending on the protein-IL combination, and the IL concentration in water. Overall, the findings indicated that some ions and some ILs were in general better for protein solubility and stability than others, such as acetate leading to poor solubility, and EAN and EAF generally leading to better protein stability than the other PILs. This study of four proteins in 10 aqueous PILs clearly showed that there are many complexities in their interactions and no clear general trend, despite the similarities between the PIL structures. This highlights the need for more and larger studies to enable the selection and optimization of PIL solvents used with biomolecules.

## Introduction

Proteins are macromolecules made up of sequences of amino acid sub units. The folding of these amino acid chains determines the proteins three-dimensional structure and functionality. The hydrophilic and hydrophobic characteristics of amino acids are important contributors to protein shape, folding and solubility. The folding of proteins are based on their amino acid sequence, and many proteins easily unfold outside of their native environment, and then can refold, mis-fold, or aggregate (Fernández and Scott, 2003). One of the major problems in stabilizing proteins is that they can easily denature and form aggregates during the process.

Previously, it was considered that non-aqueous solvents make proteins insoluble or unstable, and have therefore not been used extensively (Rariy and Klibanov, 1997). However, while polar organic solvents are generally detrimental to proteins, studies have shown that proteins can be stable in some solutions containing polar molecules, such as glycerol, which has been shown to have a stabilizing effect on lysozyme (Rariy and Klibanov, 1997; Paciaroni et al., 2002; Cinelli et al., 2004; Gögelein et al., 2011). In addition, non-polar solvents, such as cyclohexane, can be tolerated by proteins at high concentrations (Pace et al., 2004).

Ionic liquids (ILs) have emerged as interesting solvents for biological molecules, with a broad range of polar and non-polar ILs appearing to be beneficial for protein stability (Rodrigues et al., 2014). ILs are highly tailorable solvents, which allows many solvent properties to be modified and functional groups to be included. For example, their tailorable hydrophobicity makes them potentially useful as solvents for proteins which have poor solubility in water. Due to the tailorability of ILs it is feasible that these solvents could be designed to have either a stabilizing or destabilizing effect on proteins, depending on what is needed for a specific application. This has been shown for aqueous solutions containing ILs, where depending on the IL, the protein lysozyme can be stabilized (Zhao, 2016), crystallized(Niedzialkowska et al., 2016), denatured(Ortore et al., 2009), or formed into Amyloid fibrils(Cao et al., 2004; Byrne and Angell, 2009). However, the high tailorability of ILs also means that there are many possible combinations of IL cations, anions and proteins, along with water concentrations or other additives. It is unclear whether there are particular ILs which will be beneficial for classes of proteins, or whether each protein will need the solvent optimized individually. Consequently, it is necessary to build up systematic knowledge of proteins in IL containing solvents, to enable the design of IL solvents for specific proteins.

Protic ionic liquids (PILs) have been used in this study due to the ease of modifying their structure and properties, including their polarity, cation alkyl chain length, anion nucleophilicity, hydrophobicity, pH, and ionicity. PILs are synthesized by proton transfer from a Brønsted acid to a Brønsted base with equal molar ratios (Greaves and Drummond, 2008). There are previous studies which have shown the effect of certain PILs on protein stability, with the vast majority having the PILs present in aqueous solutions. Ethylammonium nitrate (EAN) was one of the first ionic liquids trialed for potentially being capable of stabilizing proteins, as it has some similar characteristics to water, such as being able to form a hydrogen bonded network (Garlitz et al., 1999), high polarity, and the ability promote amphiphile self-assembly (Evans et al., 1982). More recent studies on hen egg white lysozyme (HEWL) in EAN and triethylammonium triflate (TEATf) solutions have shown dissolution and inhibition of amyloid fibrils formation (Byrne and Angell, 2009). However, limited work has been done to understand the secondary structural changes of proteins in PILs, which PILs are optimal, the role of the cation and anion, or what PIL-water concentrations are best.

A variety of aqueous aprotic ILs has also been explored for their ability to stabilize proteins. Similar to PILs, it appears that there is a significant variation in protein stability depending on the specific protein, ionic liquid, and water content. For example, the structural stability of cytochrome c (at 50 mM) was studied using temperature-induced FTIR spectroscopy in a few biocompatible IL solutions with 20 mg/ml of 0.5–3 M IL in water. The ILs contained dicyanamide, saccharinate, or dihydrogen phosphate (dhp) anions paired with a butylmethylpyrrolidinium cation, or choline chloride (Fujita et al., 2005). It was found that solutions containing choline dhp showed no changes in protein structure during exposure to temperatures of 110 °C, providing long term thermal stability of the protein(Fujita et al., 2005). FTIR analysis of lipase in ILs containing dicyanimide, alkylsulfate, nitrate and lactate anions paired with [BMIm][BF_4_] showed the secondary structure was maintained, with the conformation of the enzyme closely resembling the native one (Madeira Lau et al., 2004). There has been evidence showing that ILs can stabilize RNase at pH 7.4, with previous experiments identifying certain beneficial ions like 1-alkyl-3-methylimidazolium,N,N-dialkylpyrrolidinium and tetraalkylammonium for protein stability, whereas [PF_6_] and [BF_4_] were detrimental (Weingärtner et al., 2012).

Previously, we reported on the activity and conformation of hen egg white Lysozyme in aqueous solutions of ethylammonium nitrate (EAN), ethylammonium formate (EAF), ethanolammonium formate (EtAF), ethanolammonium nitrate (EtAN), aqueous solutions of molecular solvents, and a selection of highly concentrated salts (Wijaya et al., 2016, 2018). Of these it was observed that EAN and EtAF led to maximum lysozyme activity, comparable to conventional salts, whereas EAF and EtAN did not. Similarly, lysozyme activity was maintained in up to very highly concentrated PIL solutions for EAN and EtAF, whereas EAF and EtAN led to denaturation and aggregates at higher PIL concentrations. The maximum activity of lysozyme was lower in the molecular solvents than in EAN or EtAF solutions. Interestingly the two PILs of EAN and EtAF did not share common ions, indicating that the combination of IL ions is perhaps more important than which ions are present. It also clearly showed that comparable activities can be obtained using PILs compared to conventional salts, but without any salt solubility limit due to the miscibility of these PILs with water (Wijaya et al., 2016).

**Figure 1:**
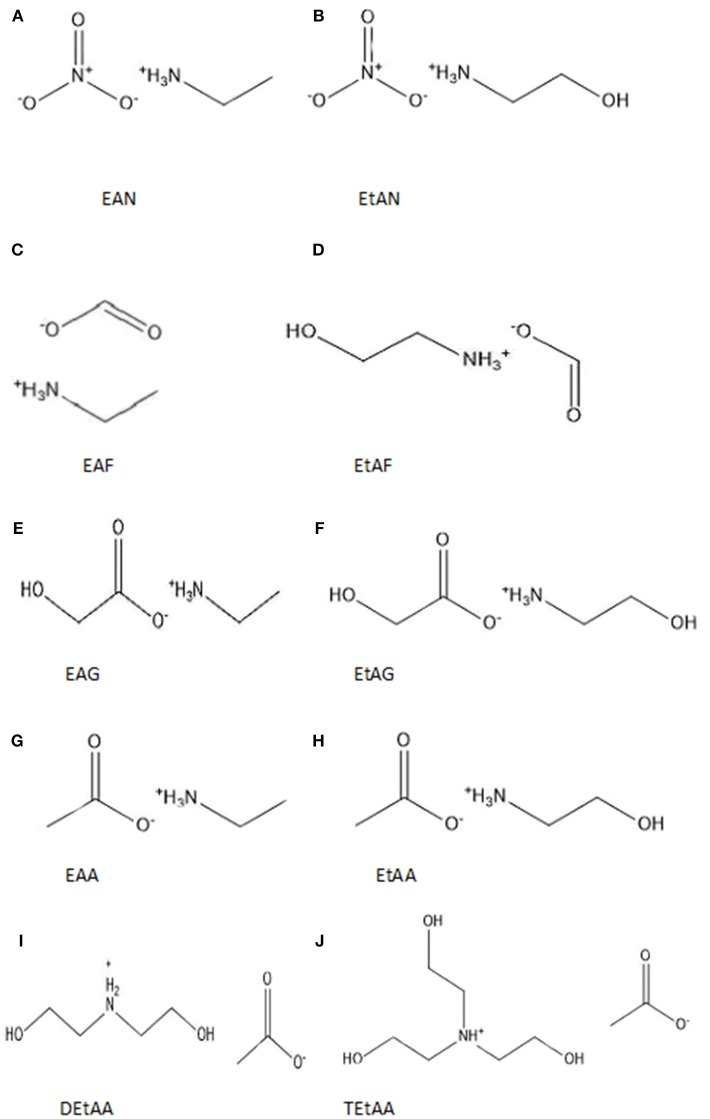
Chemical structures and abbreviations of the 10 PILs used in this investigation **(A)** ethylammonium nitrate (EAN), **(B)** ethanolammonium nitrate (EtAN), **(C)** ethylammonium formate (EAF), **(D)** ethanolammonium formate (EtAF), **(E)** ethylammonium glycolate (EAG), **(F)** ethanolammonium glycolate (EtAG), **(G)** ethylammonium acetate (EAA) **(H)** ethanolammonium acetate (EtAA), **(I)** diethanolammonium acetate (DetAA), and **(J)** triethanolammonium acetate (TetAA).

In this work we have used PILs as tuneable solvents to investigate the effect of the solvent environment on protein stability. We have investigated the secondary structure of four proteins in aqueous solutions of 10 PILs. This significantly extends upon our previous work and aims at exploring the role of individual ions and the combinations of cations and anions. The chemical structures of the 10 PILs are provided in Figure 1 and have been selected to cover short alkylammonium cations with and without hydroxyl groups, and with primary, secondary and tertiary structures. These were paired with nitrate, formate, acetate, and glycolate anions. A selection of properties of the neat ILs are provided in Table S1 of the ESI, including their molecular weight, melting point, viscosity and density. The proteins selected were lysozyme, trypsin, α-amylase, and β-lactoglobulin. The proteins were selected to provide a variety of sizes, shapes, iso electric points (pI), hydrophobicity/hydrophilicity and secondary structures. The secondary structures of the proteins in each solution were characterized using Fourier Transform Infrared Spectroscopy (FTIR).

## Experimental Methods

Lysozyme from chicken egg white (EC 3.2.1.17), trypsin from bovine pancreas (EC 3.4.21.4), α-amylase from *Bacillus licheniformis* (EC 3.2.1.1) and β-lactoglobulin from bovine milk (EC 2329289) were obtained from Sigma Aldrich. Ethylamine (Sigma-Aldrich, 70 wt%), ethanolamine (Chem Supply, 99%), diethanolamine (Chem Supply, 98%), triethanolamine (Chem Supply, 99%), nitric acid (Chem supply, 70% w/w), acetic acid (Chem Supply, 99%), glycolic acid (Chem Supply, 99%) and formic acid (Merck, 98–100%) were used without further purification for the synthesis of the PILs.

The PILs were synthesized by slowly adding equimolar amounts of the acid to the base. The solution was continuously stirred, and the temperature maintained below 10°C using an ice bath. Small portions of methanol were added to the amine for the PILs which otherwise would form a solid during the reaction. Methanol and excess water were removed by drying under vacuum at >0.01 Torr on a rotary evaporator. Further drying was carried out using a LabconcoFreeZone® 4.5 Liter Freeze Dry System, for up to 24 h. The water content of the PILs was determined by Karl Fischer Titration, using a Mettler Toledo DL39 Karl Fischer coulometer, and the PILs all had <2 wt% water. Aqueous PIL solutions were prepared for each PIL with 5, 10, 20, 30, and 50 mol% of PIL ions present. The wt% for each of these compositions is provided in Table S2 of the ESI.

Samples of the proteins in the PIL-water solutions were prepared using 20 mg/ml of each of these four proteins in each of the 50 aqueous PIL solvents, along with water for comparison. The proteins were added to 1 ml Eppendorf tubes in a powder form, followed by addition of the solvents. The proteins were dissolved by gently vortexing for 10–30 s. Samples which did not dissolve were then further vortexed for 1–5 min and the protein state checked after 24 h.

Fourier transform infrared (FTIR) spectra were recorded using a Perkin-Elmer Frontier MID/FAR IR instrument with a diamond ATR (attenuated total reflectance) crystal. Protein concentrations of 20 mg/ml were used for all samples, and the samples were left to equilibrate for 1 h prior to measurements, with all measurements being made in the next 1 h. Each sample was characterized using 32 scans with a resolution of 2 cm^−1^ over the range of 400 cm^−1^ to 4000 cm^−1^. FTIR spectra of each solvent, without the protein present, were acquired under the same conditions and used for solvent subtraction.

## Results

Solutions of each of the 10 PILs, shown in Figure 1, were prepared with 5, 10, 20, 30, and 50 mol% of the PIL ions in water, leading to 50 individual aqueous PIL solvents (the corresponding concentrations in wt% are provided in Table S2 of the ESI). These concentrations are well-outside the concentration ranges used for conventional salts and buffers for protein stability work, and beyond the solubility of many salts in water. The protein stability of four globular proteins, lysozyme, trypsin, α-amylase and β-lactoglobulin, were investigated in each of the aqueous PIL solutions. These proteins were selected to explore a diverse range of enzymatic globular proteins. The size and isoelectric point for these four proteins is provided in Table 1.

**Table 1:** Size and isoelectric point, pI, of the four proteins.

**Protein**	**Size (kDa)**	**Isoelectric point (pI)**
HEW Lysozyme	14.3 (Canfield, 1963)	11.35 at ionic strength 0.1 (Wetter and Deutsch, 1951)
Trypsin from bovine pancreas	23.8 (Cunningham, 1954)	10.5 (Buck et al., 1962)
α-Amylase from *Bacillus licheniformis*	62.7 (Morgan and Priest, 1981), 58.0 (Ivanova et al., 1993; Rao et al., 2002), 64.0 (Kim et al., 1992)	5.4 (Kim et al., 1992), 6.9 (Ivanova et al., 1993), 7.18 (Rao et al., 2002)
β-Lactoglobulin from bovine milk	18.3 (Ding et al., 2011)	5.1 (Engelhardt et al., 2013)

Visual observations were made of the resulting solutions to identify which solutions led to the 20 mg/ml proteins dissolving, forming a gel, or not fully dissolving. These observations have been provided in Tables 2–5 for lysozyme, trypsin, α-amylase, and β-lactoglobulin, respectively. The open squares represent the protein-solvent conditions where the protein did not fully dissolve, and crosses where the addition of the protein caused the solution to form a gel.

**Table 2:** Summary of the physical state and secondary structure of lysozyme in the aqueous PIL solvents.

**PILS**	**PIL concentration in mol%**				
	**5**	**10**	**20**	**30**	**50**
EAN	♦	♦	♦	♦	♦^*^
EAF	♦	♦	♦^*^	^*^	^*^
EAG	♦	♦	♦	♦	^*^
EtAN	♦	♦	^*^ ×	×	×
EtAF	♦	♦	♦	♦	♦
EtAG	♦	♦	♦^*^	^*^	□
DEtAA	^□^	^□^	^□^	^□^	^□^
TEtAA	♦	♦^*^	♦^*^	□	□
EAA	□	□	□	□	□
EtAA	□	□	□	□	□

♦ FTIR consistent with native state.

♦^*^ FTIR small changes.

* Changes in the secondary structure.

□ Visually observed to not fully dissolve.

× * Viscous gel*.

**Table 3:** Summary of the physical state and secondary structure of α-amylase in the aqueous PIL solvents.

**PILS**	**PIL concentration in mol%**				
	**5**	**10**	**20**	**30**	**50**
EAN	♦^*^	♦^*^	^*^	□	□
EAF	×	×	×	×	×
EAG	♦^*^	♦^*^	♦^*^	^*^	×
EtAN	♦	×	×	×	×
EtAF	□	□	□	□	□
EtAG	♦^*^×	×	×	×	×
DEtAA	□^$^	□	□	□	□
TEtAA	♦	♦	♦	□	□
EAA	□^$^	□	□	□	□
EtAA	□^$^	□	□	□	□

♦ FTIR consistent with native state.

* Changes in the secondary structure.

□ Visually observed to not fully dissolve.

× Viscous gel.

$ * Formed a gel after 5–10 min*.

**Table 4:** Summary of the physical state and secondary structure of trypsin in the aqueous PIL solvents.

**PILS**	**PIL concentration in mol%**				
	**5**	**10**	**20**	**30**	**50**
EAN	*	*	*	*	*
EAF	*	*	*	*	*
EAG	♦^*^	♦^*^	♦^*^	*	□
EtAN	♦	♦	* ×	♦^*^×	×
EtAF	♦	♦	♦	*	*
EtAG	♦	*	□	□	□
DEtAA	□	□	□	□	□
TEtAA	♦^*^	♦^*^	♦^*^	*	*
EAA	□	□	□	□	□
EtAA	□	□	□	□	□

♦ FTIR consistent with native state.

* Changes in the secondary structure.

□ Visually observed to not fully dissolve.

× * Viscous gel*.

**Table 5:** Summary of the physical state and secondary structure of β-lactoglobulin in the aqueous PIL solvents.

**PILS**	**PIL concentration in mol%**				
	**5**	**10**	**20**	**30**	**50**
EAN	♦	♦	♦	□	□
EAF	□	□	□	□	□
EAG	♦^*^	♦^*^	♦^*^	*	□
EtAN	*	*	×	×	×
EtAF	♦	♦	♦	♦	□
EtAG	♦^*^	♦^*^	♦^*^	♦^*^	□
DEtAA	□ $	□	□	□	□
TEtAA	*	*	*	□	□
EAA	□ $	□	□	□	□
EtAA	□ $	□	□	□	□

♦ FTIR consistent with native state.

* Changes in the secondary structure.

□ Visually observed to not fully dissolve.

× Viscous gel.

$ *formed a gel after 5–10 min*.

In general, the proteins dissolved well in these aqueous PIL solutions, with the exceptions of the acetates. All four proteins did not fully dissolve in any of the EAA, EtAA, or DEtAA containing solutions. In contrast, all four proteins were soluble in the bulkier TEtAA, for TEtAA concentrations of 5, 10, and 20 mol%. For the non-acetates, α-amylase was not soluble in any of the EtAF solutions, and β-lactoglobulin was not soluble in any EAF solutions.

For a few protein-IL combinations, the proteins fully dissolved and then formed a very viscous solution, which has been referred to as a gel. The gelation of proteins is highly dependent on the solvent condition, and typically occurs when proteins partially unfold and form cross links between protein molecules. Globular proteins require higher concentration for gel formation, with interactions at low concentrations tending to occur within molecules rather than between molecules, and hence the gel network is not formed (Ziegler and Foegeding, 1990). It has been shown that some proteins like α-lactalbumin do not exhibit gelling properties because of the nature of their poly-peptide linkage (Paulsson et al., 1986). Gel formation occurs when partially unfolded proteins develop uncoiled polypeptide segments which interacts at certain points to form a three dimensional cross-linked network, and with gels forming if there is sufficient cross-linking present (Hill et al., 1998). All four proteins formed a gel in EtAN solutions immediately when the concentration of EtAN was 20 mol% or higher, as well as at 10 mol% for α-amylase in EtAN. In addition, α-amylase formed a gel in EAF and EtAG for all PIL concentrations trialed. This is consistent with what has previously been reported with gelation more likely for proteins containing more hydrophobic groups, and α-amylase was the most hydrophobic of the proteins used (Zayas, 1997). In addition, the ability of EtAN to act as a hydrogen bond donor or acceptor is likely to contribute to the gel formation, with perhaps the strong nitrate anion contributing in destabilizing the protein and exposing the hydrophobic core.

**Figure 2:**
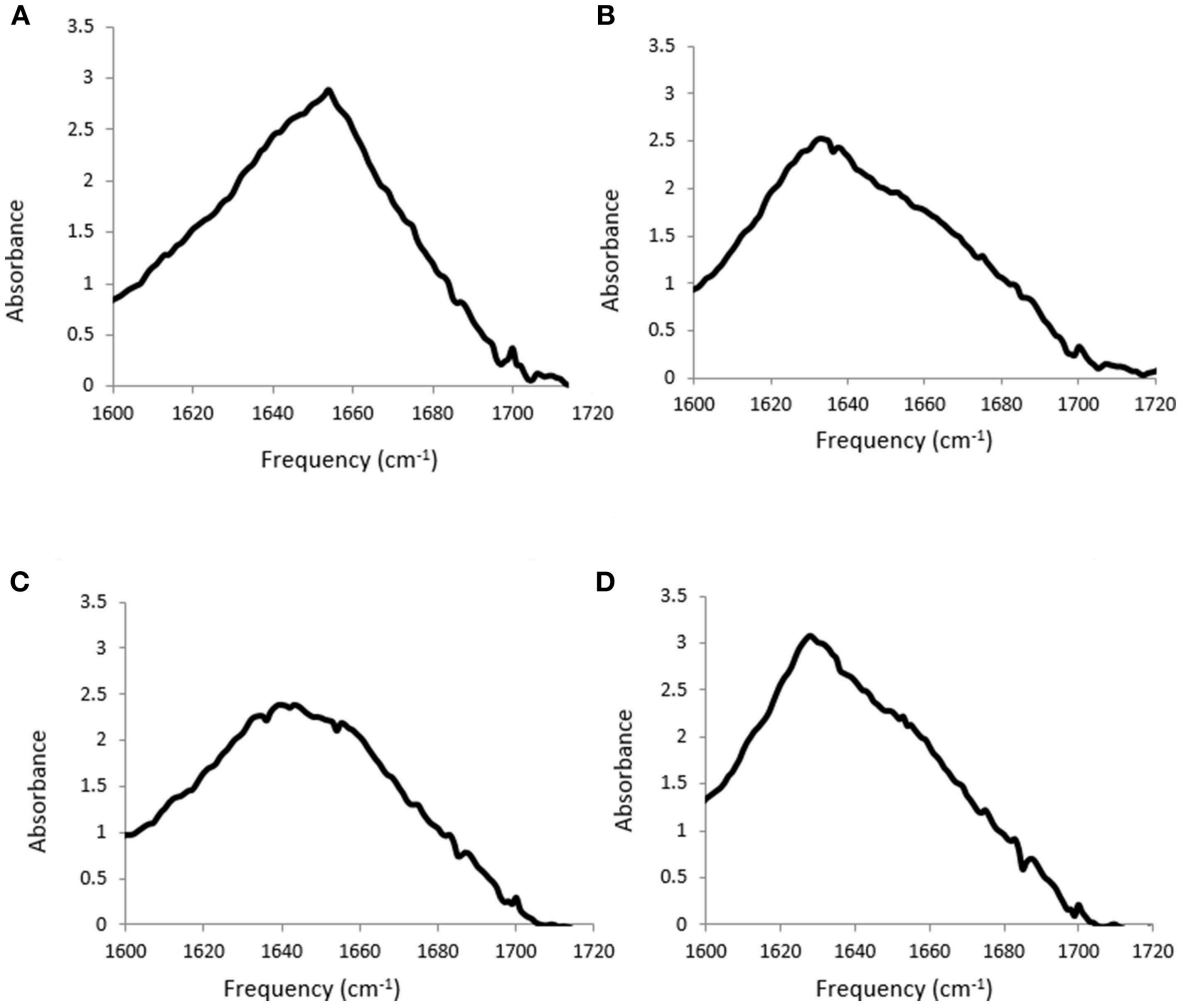
Amide I band from FTIR spectra for **(A)** lysozyme, **(B)** trypsin, **(C)** α-amylase and **(D)** β-lactoglobulin at 20 mg/ml in water.

FTIR spectroscopy was used to investigate the secondary structure of the proteins in these aqueous PIL solutions. FTIR is sensitive to protein structural changes, and unlike circular dichroism it is useable for these solutions, which can be considered as extremely concentrated salt solutions. There are two broad absorption bands observed for proteins using FTIR, conventionally called amide I and amide II bands, at wavenumbers 1,700–1,600 cm^−1^ and 1,600-1,500 cm^−1^, respectively. The Amide I band is more commonly used for characterizing the secondary structure, and is due to C = O stretching vibrations of the peptide bonds, which are modulated by the secondary structure (α-helix, β-sheet, etc.). The sum of the spectral contributions from these features lead to a broad band with overlapping sub spectra, and the frequency associated with the main protein secondary structural features are provided in Table 6. The Amide I band for each of the four proteins in water is shown in Figure 2.

**Table 6:** The FTIR frequency of the dominant secondary structural components of proteins contributing to the Amide I band (Creamer et al., 1983; Knubovets et al., 1999).

**Assignment**	**Frequency (cm^**−1**^)**
β-sheet	1,626–1,640
Random structure	1,640–1,651
α-helix	1,650–1,657
β-turn	1,655–1,675

**Figure 3:**
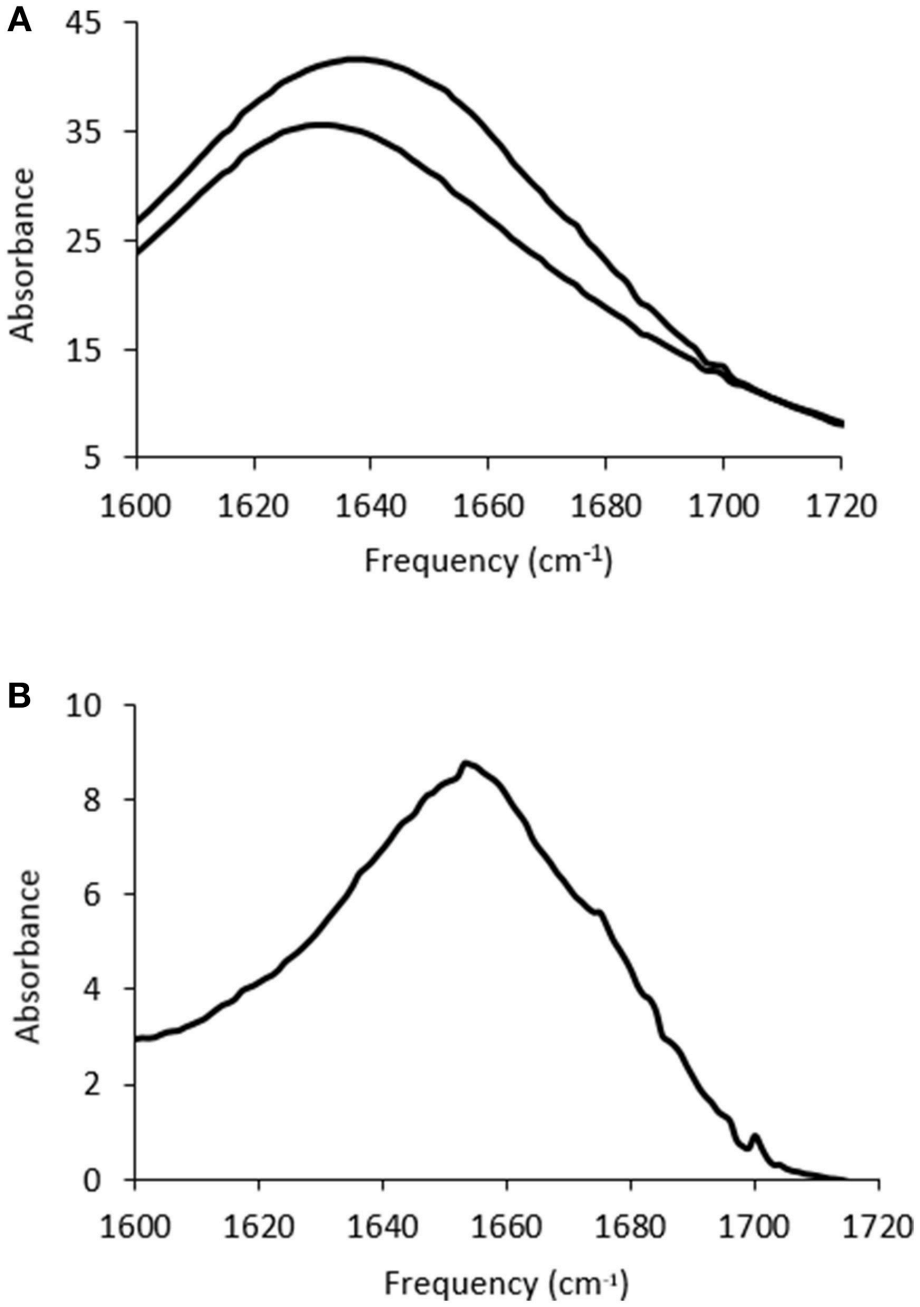
FTIR spectra for **(A)** 5 mol% EAN in water (lower trace) and 20 mg/ml of lysozyme in this solution (upper trace), and **(B)** the Amide I band after solvent subtraction for the spectra in **(A)**.

The solvent contribution was subtracted for each sample, which is difficult due to the ILs containing features which overlap with the Amide I band. An example is shown in Figure 3 for lysozyme in 5 mol% EAN, with the FTIR spectra of the solvent, and protein in solvent, before solvent subtraction shown in Figure 3A, and the resulting spectra after subtraction in Figure 3B. Consequently, due to the solvent contributions in the wavenumber region of interest these measurements require identical solvents to be used for the solvent blank and protein containing solution, and identical volumes for both solutions. In addition, for it to be considered a good background subtraction, the baseline needed to be straight between 1,800–1,500 cm^−1^ and 2,000–1,750 cm^−1^, consistent with the literature methods (Kumosinski and Farrell, 1993; Wijaya et al., 2016).

Subtracted FTIR spectra were obtained for all the protein-aqueous IL solutions, where the protein dissolved. These are provided in Figures S1–S25 of the ESI. Spectra were also acquired for lysozyme in a solution of EAN at 10 mol% at 1, 2, and 3 h after mixing which showed minimal variation between spectra, and these are provided in Figure S26 of the ESI. There was a significant variation in the behavior of the secondary structure for each protein-IL combination, varying between negligible change and major disruption of the secondary structure. A summary is provided in Tables 2–5 for which solvents retained the secondary structure of the protein consistent to what was seen in water (♦). For some spectra there was only minor changes to the secondary structure, and these have been denoted by an additional ^*^ next to the ♦.

**Figure 4:**
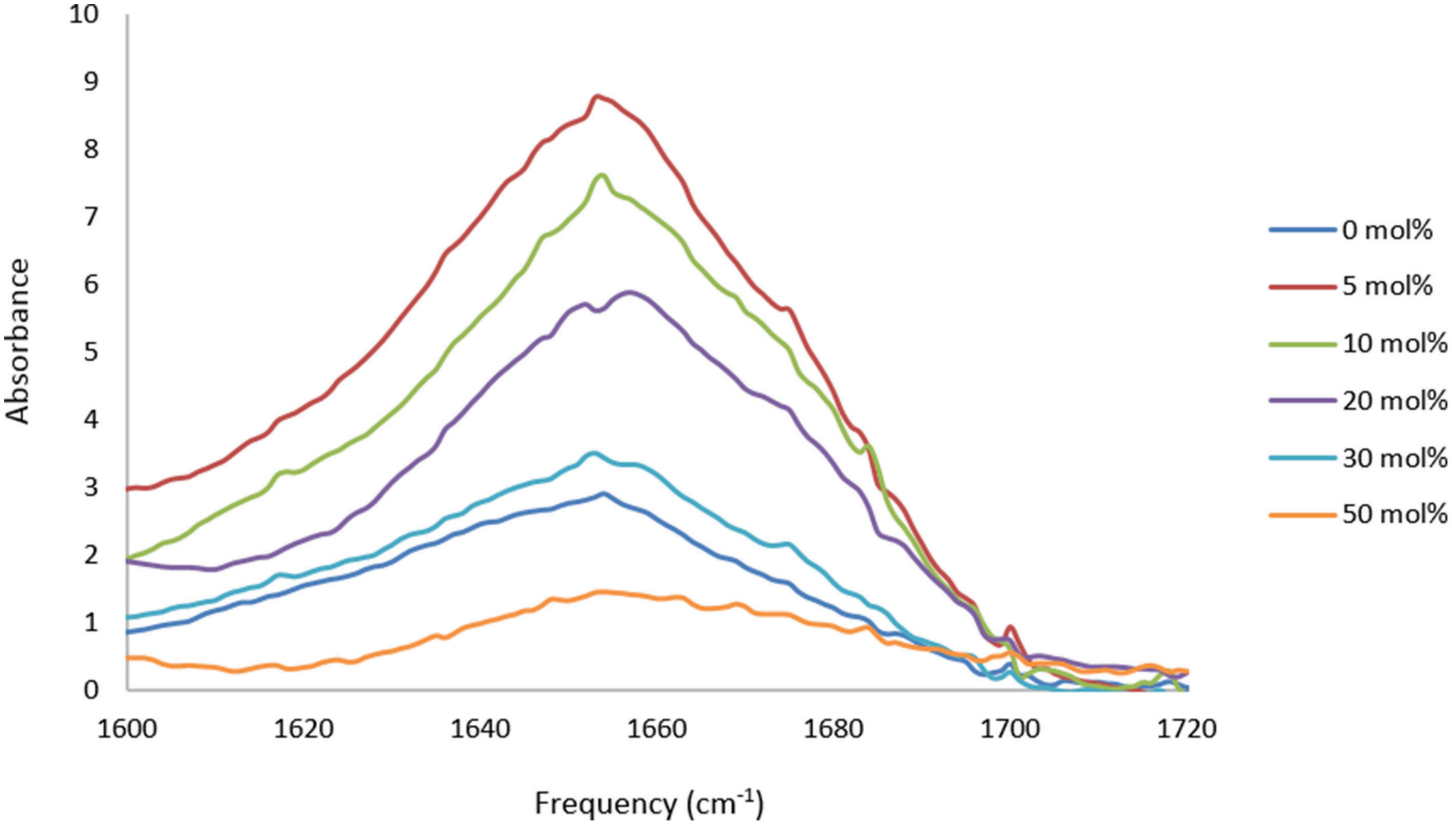
Amide I region of the FTIR spectra of lysozyme in aqueous solutions of 5, 10, 20, and 30 mol% of EAN.

The proteins in all the other PIL-water series, where they were soluble, showed evidence of changes in their secondary structures at either some, or all, PIL concentrations. It is evident from Tables 2–5 that the solvent conditions which supported the proteins in having FTIR spectra, consistent with the native state, were substantially different for the four proteins. As shown in Tables 2–5 there were only two protein-IL combinations where the protein retained its secondary structure across all concentrations of the ILs. These were lysozyme in EAN and EtAF. The amide I band for lysozyme in EAN solutions is shown in Figure 4, and it is apparent that there was little change in the secondary structure from 0 to 50 mol% of EAN. However, when the EAN concentration was 50 mol% there appeared to possibly be a small increase in the proportion of β turn present. There was good stability for lysozyme in solutions of 5 mol% of many of the PILs (EAN, EAF, EAG, EtAN, EtAF, EtAG and TEtAA), and at 10 and 20 mol% for many of these. For these solutions, the dominant peak in the FTIR spectra was consistent with the alpha helix structures being retained.

**Figure 5:**
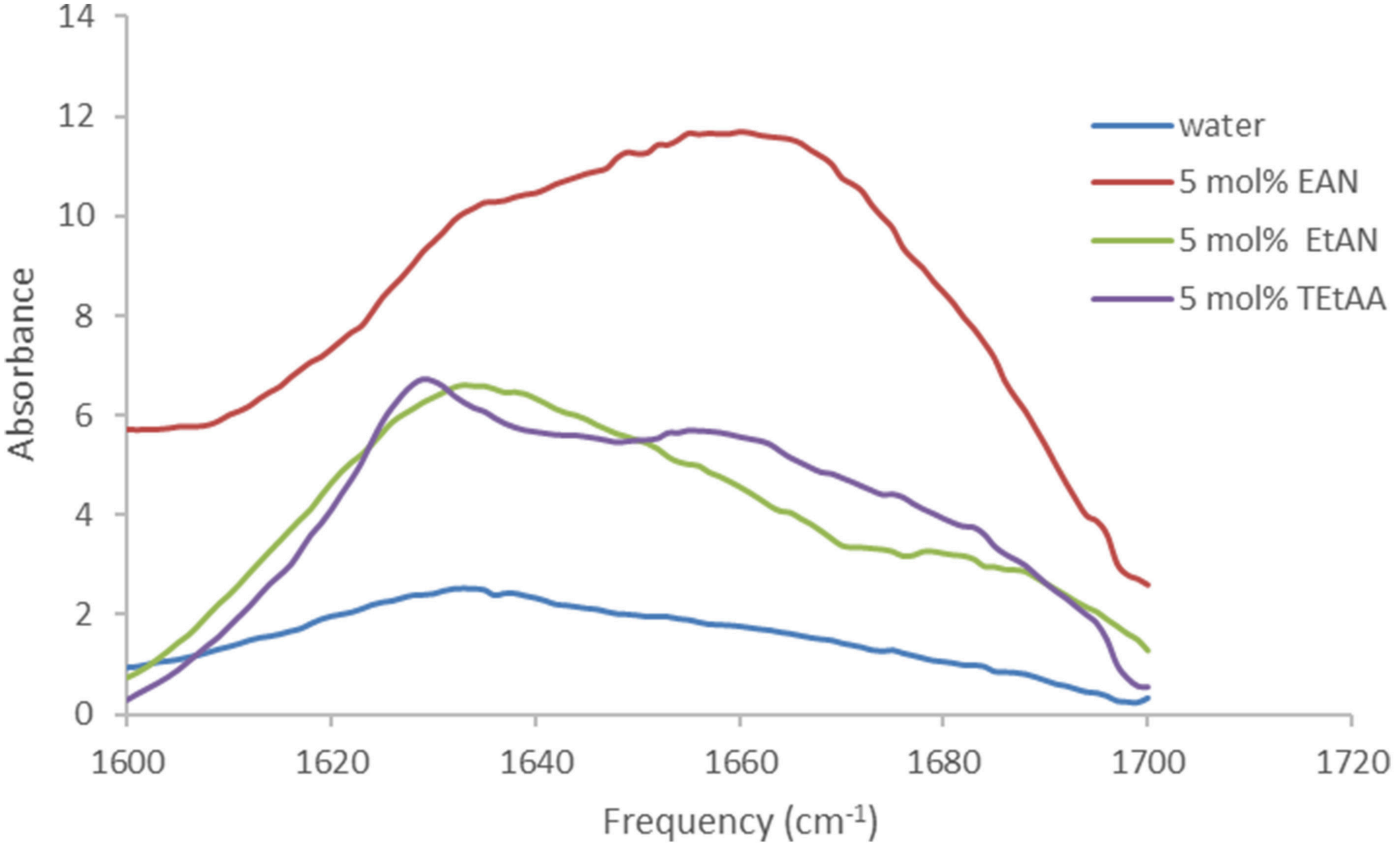
Amide I region of the FTIR spectra of trypsin in aqueous solutions of 5 mol% of EtAN and TEtAA.

The spectra for trypsin in aqueous solutions containing 5 mol% of EtAN or TEtAA are provided in Figure 5. At this lowest PIL concentration Trypsin retained its secondary structure in EtAN, shown in Figure 5, and also in EtAF and EtAG. However, for the PILs of EAG and TEtAA there were some changes to the secondary structure, consistent with a small increase in beta structure, and the spectra for Trypsin in 5 mol% TEtAA is shown in Figure 5. The secondary structure of Trypsin in 5 mol% EAN and EAF underwent significant changes, showing large proportions of beta structures.

**Figure 6:**
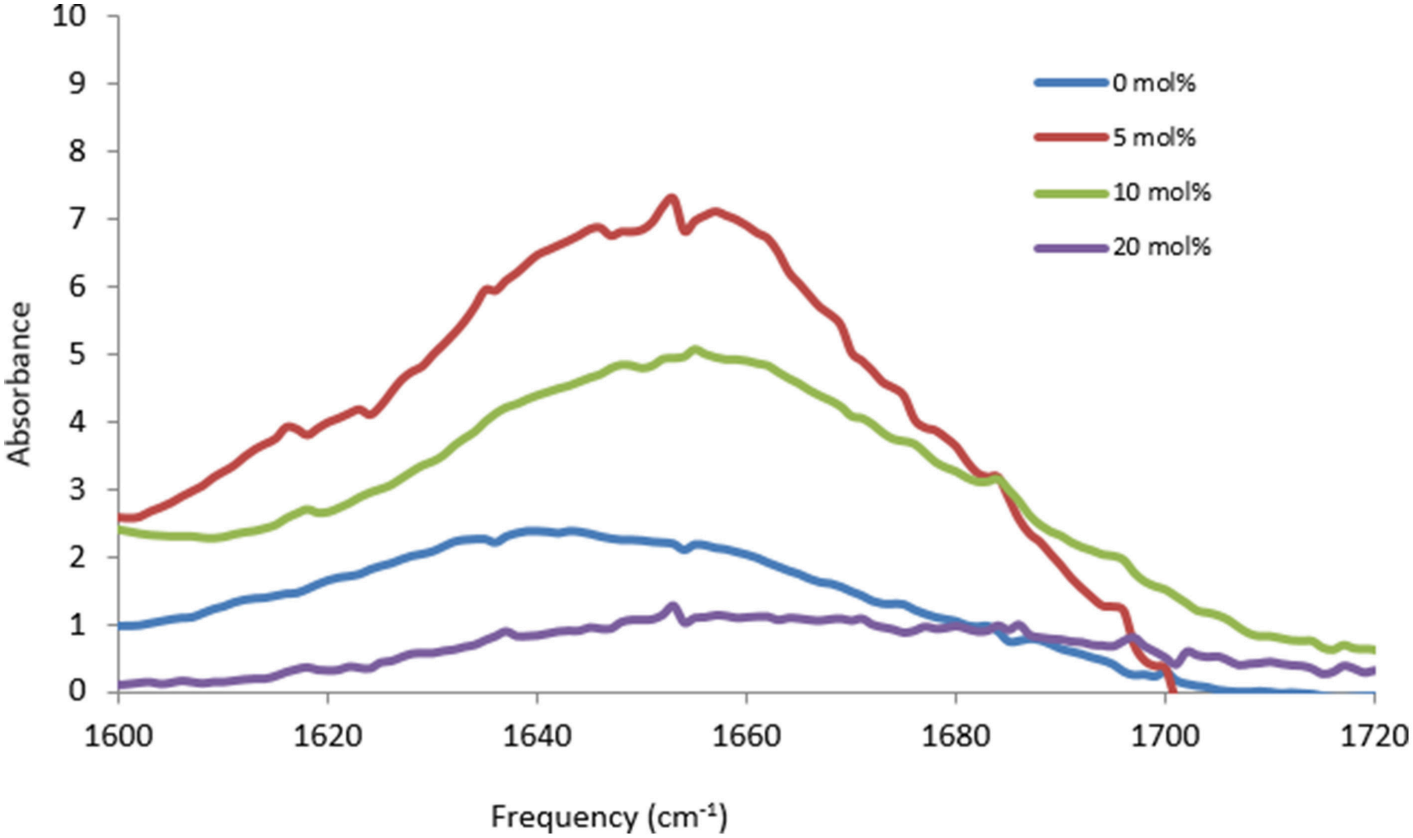
Amide I region of the FTIR spectra of α-amylase in aqueous solutions of 0, 5, 10, and 20 mol% of EAN.

Based on the FTIR spectra, the secondary structure of amylase was altered in many of the PIL containing solvents. An example is shown in Figure 6 for amylase in aqueous EAN solvents. At 5 and 10 mol% there were some minor changes in the spectra, whereas at 20 mol% there appears to be a major loss of alpha helix structures and a large gain in beta structures. For higher PIL concentrations of 30 or 50 mol % EAN the amylase did not dissolve.

It was evident that the dominant change in secondary structure, away from native structure for all of four proteins in the PIL-solutions, was an increase in the proportion of beta structures present. These beta structures then led to the aggregation and gelation of the proteins, likely through disruption to the intermolecular interactions between hydrophobic regions, leading to exposed hydrophobic groups. These exposed hydrophobic surfaces can interact between proteins, and lead to either amorphous or fibril aggregates.

## Discussion

The stability and solubility of proteins are important factors for the pharmaceutical industry (Brader et al., 2015). The stability of proteins depend on environmental properties such as pH, ionic strength, and the presence of buffers/salts. Protein stability in an ionic solution is highly complex, and depends on the protein-ion, ion-ion, ion-water, protein-water and water-water interactions. The ions can modify the local structure of water molecules, increasing or decreasing how structured it is, and hence change how entropically favorable it is for proteins to be dissolved. It was evident from this investigation that the protein stability in concentrated aqueous ILs solutions varied substantially depending on the protein-IL combination.

There have been several efforts to minimize protein aggregation, enhance stability and improve protein crystallization, by utilizing appropriate screening techniques (Kheddo et al., 2014; Liu et al., 2016; Zhou et al., 2018). Here we used visual observations and FTIR as suitable screening methods for the characterization of protein stability. These relatively fast techniques enabled the investigation of four proteins in 50 different IL-water solutions. In this work, the ion concentrations were much greater than those used for conventional protein stability solutions in buffers, with concentrations from 5 to 50 mol% of ILs. This makes it difficult to compare with much of the conventional literature using aqueous buffers. In addition, the literature discussions surrounding salting in and salting out effects are valid for low concentrations of ions, whereas at higher concentrations previous studies have indicated that most ions behave with a combination of weak electrostatic repulsion and other attractive interactions, causing proteins to aggregate (Yang et al., 2010).

The properties of the ions have a significant influence on their interactions. Consistent with previous studies, the anion in our series of ILs had a dominant effect on the protein stability. This is attributed to its hydrogen bond forming capability, nucleophilicity properties, and ability of the anion to strongly interact with the enzyme, thus leading to conformational change in the structure and activity of the enzyme (Zeuner et al., 2011).

Previously, we showed that the combination of ions plays an important role in protein stability for lysozyme in PIL solutions of EAN, EtAN, EAF, EtAF. For these PILs, the data correlated well with the “Collins law of matching water affinity” (Collins, 2004) with good protein stability occurring where both ions could be considered either kosmotropic or chaotropic, but poor if they were different (Wijaya et al., 2016). In an non-IL study it was also found that when both anions had similar kosmotropic or chaotropic behavior, proteins usually were stabilized by a kosmotropic anion and chaotropic cation and destabilized by the opposite, for IL concentrations up to 1M (Yang et al., 2010). In this study we clearly see that the combination of ions was crucial; however, they behaved differently for each protein and many trends were not consistent across all proteins. The findings from this current study showed that the trends for those four PILs with Lysozyme did not extend to the other proteins. However, it was apparent that some ILs, such as EAN and EtAF were generally more favorable, and that some ions, such as acetate, were not. This highlights the importance of larger studies for proteins in ILs which will enable more robust structure-property relationships to be developed.

The acetate ion was the least favorable ion of the anions used in this investigation. It was observed that even at low concentrations all four proteins were insoluble in ethyl-, ethanol- and diethanolammonium acetate solutions, though all were soluble in the presence of triethanolammonium acetate at lower concentrations, with lysozyme and amylase retained in a native state. Acetate ions have previously been seen to decrease the solubility of proteins, though it depends on the cations they are paired with. For example, ILs containing acetate are weak stabilizers when paired with the more hydrophobic imidazolium or phosphonium cations (Wijaya et al., 2018), while acetate paired with triethylammonium has been shown to be a strong stabilizer (Attri et al., 2011). In this study we observed that the hydroxyl version of the tertiary ammonium cation, TEtA cation, also appears to enhance protein solubility, and stability for some proteins when paired with acetate. In contrast the acetate salts of the primary and secondary cations of EtA and DEtA led to poor protein solubility. The hydration number and ion-water interactions, as well as ion-pair formation, are crucial factors for how the ion and water in the solvent will interact with proteins. These depend on the size, shape and charge density of ions. Ions can be widely classified into either kosmotropes or chaotropes according to their ability to bind to water (Manincelli et al., 2007). Acetate can be considered a kosmotropic anion, and likely to have strong interactions with multiple water molecules. Considering the very high proportion of ions in these solutions, this may result in the majority of the water present to interact with acetate anions, and therefore either having fewer, or modified water interactions with the proteins, potentially leading to the poor solubility.

The comparison of the protein stability in these IL solutions to conventional aqueous salts is difficult, particularly because of the much higher concentrations of ions used here. One common ion description is of ions leading to salting in or salting out of proteins, but this is only meaningful for ion concentrations up to 1 M (Schröder, 2017). In this study, the ion concentrations are much greater than those used for conventional protein stability solutions in buffers. In terms of salting in and salting out, all the ions will all behave the same at these higher concentrations, with the ionicity effect leading to weak electrostatic repulsion contrasted with opposing attractive interactions, decreasing protein solubility, and increasing the preference to aggregate (Yang et al., 2010).

The effect of ions on proteins can be classified for many aqueous systems by using the Hofmeister series (Timasheff, 1993; Hribar et al., 2002; Pegram and Record, 2007; Yang et al., 2010; England and Haran, 2011; Schröder, 2017). In this series, cations and anions are ordered based on their tendency to solubilize, or stabilize proteins. This series definitely does not apply to protein stability in all IL solutions, with different studies showing that IL ions can follow the Hofmeister series, follow reverse Hofmeister series, or are anomalous to the Hofmeister series (Warren et al., 1966; Yang, 2009). Similarly, in this study we have clearly seen that there is not a consistent trend in the IL ions for protein stability or solubility across all four proteins, or with the Hofmeister series. This indicates that there are more complex interactions present, and that the “observational” Hofmeister series is not sufficient to classify ILs for protein stability (Mazzini and Craig, 2018). Alternative methods have recently been discussed by Mazzini and Craig, and better alternatives involve using ion water affinity and ion sizes, and considering specific-ion effects (Mazzini and Craig, 2018).

The isoelectric point, pI, of these proteins is given in Table 1, and these proteins were selected due to the broad range of pI's. The protein stability in these ILs indicated that the pI of the proteins had an effect. Trypsin and lysozyme had higher pI's and were stable in a broader range of these IL solvent conditions. In contrast, α-amylase and β-lactoglobulin had pI's below 7 and displayed generally poorer stability and solubility in the IL solutions, forming aggregates or precipitates in many of the solvent conditions trialed. This may be due to the pI of these proteins being closer to the pH of the solvents. In aqueous solutions, the pH scale is routinely used to measure proton activity. However, the conventional pH scale is not valid in neat ionic liquids or highly concentrated ionic liquid solutions such as these.

## Conclusions

This study shows that the nature of proteins and the combination of ions are all important in stabilizing proteins. The changes in the secondary structure of proteins using FTIR spectroscopic analysis, proved an efficient tool to quantify wide arrays of protein-ion combinations. Understanding protein stabilization is a vast process involving different solvent-protein properties. In this work, we identified that some of the PILs containing nitrates, formates and glycolates were able to retain the secondary structure of proteins at the lower IL proportions, with poorer protein solubility at high IL concentrations. There was a significant variation over which ILs the four different proteins were stable, with generally EAN and EtAF being good co-solvents, whereas the acetate containing PILs led to poor proteins solubility. This study highlights the need for extensive studies on protein stability in IL solutions with a broader range of ILs, proteins, and IL concentrations.

## Author Contributions

RA completed the experimental work and much of the analysis and writing of the paper. TG trained and assisted RA with the experimental work and data analysis. CD assisted with interpretation of the results.

### Conflict of Interest Statement

The authors declare that the research was conducted in the absence of any commercial or financial relationships that could be construed as a potential conflict of interest.
