# Supplementary material for: FTIR Spectroscopic Study of the Secondary Structure of Globular Proteins in Aqueous Protic Ionic Liquids
Source: Front Chem. 2019 Feb 13;7:74. doi: 10.3389/fchem.2019.00074 (PMC6381012; doi:10.3389/fchem.2019.00074)
Supplement: Supplementary file 1 [file Data_Sheet_1.docx]

**FTIR Spectroscopic Study of the Secondary Structure of Globular Proteins in aqueous Protic Ionic Liquids**

Radhika Arunkumar^a^, Calum J. Drummond^a^ and Tamar L. Greaves^a*^

^a^ *School of Science, College of Science, Engineering and Health, RMIT University, Melbourne, VIC, Australia*

**Electronic Supporting Information**

**Table S1.** Molecular weight, melting point, T_m_, density, ρ, and viscosity, η, values for the neat ILs, as reported in the literature.

| **PIL** | **MW** | **T_m_ (^o^C)** | **ρ (g/cm^3^) at 27 ^o^C** | **η (cP) at 25 ^o^C** |
| --- | --- | --- | --- | --- |
| EAN | 108.1 | 9^1^ | 1.216^c^ | 32^d^ |
| EtAN | 91.11 | 51^1^ | 1.265^c^ | 113^d^ |
| EAF | 121.14 | -15^1^ | 1.039^c^ | 32^d^ |
| EtAF | 124.1 | ^a^ | 1.184^c^ | 220^d^ |
| EAG | 107.11 | ^a^ | 1.189^c^ | 1200^d^ |
| EtAG | 137.14 | 106 | ^b^ | ^b^ |
| EAA | 165.19 | 87 | ^b^ | ^b^ |
| EtAA | 209.24 | ^a^ | 1.176^c^ | 701^d^ |
| DEtAA | 105.14 | 55^2^ | 1.181^d 3^ | 5647^d 3^ |
| TEtAA | 121.14 | 47^2^ | 1.1752^e 4 supp^ | 797^e 4 supp^ |

^a^ No T_m_ detected.^1^

^b^ Ionic liquid solid at this temperature.

^c^ 27 ^o^C, ^d^ 25 ^o^C, ^e^ 35 ^o^C

**Table S2.** PIL-water compositions in wt% corresponding to each of the mol% concentrations

| **PIL** | **wt% (5 mol%)** | **wt% (10 mol%)** | **wt% (20 mol%)** | **wt% (30 mol%)** | **wt% (50 mol%)** |
| --- | --- | --- | --- | --- | --- |
| EAN | 20.8 | 25.0 | 33.3 | 41.7 | 58.3 |
| EtAN | 23.8 | 27.8 | 35.8 | 43.8 | 59.9 |
| EAF | 19.1 | 23.4 | 31.9 | 40.4 | 57.4 |
| EtAF | 18.8 | 23.1 | 31.6 | 40.2 | 57.3 |
| EAG | 21.0 | 25.1 | 33.5 | 41.8 | 58.4 |
| EtAG | 17.5 | 21.8 | 30.5 | 39.2 | 56.6 |
| EAA | 15.4 | 19.8 | 28.7 | 37.6 | 55.5 |
| EtAA | 13.2 | 17.8 | 26.9 | 36.0 | 54.3 |
| DEtAA | 21.3 | 25.4 | 33.7 | 42.0 | 58.6 |
| TEtAA | 19.1 | 23.4 | 31.9 | 40.4 | 57.4 |

**Figure S1.** Amide I region of the FTIR spectra of lysozyme in aqueous solutions of 0, 5, 10, 20, 30 and 50 mol% of EAN.

**Figure S2.** Amide I region of the FTIR spectra of trypsin in aqueous solutions of 0, 5, 10, 20, 30 and 50 mol% of EAN.

**Figure S3.** Amide I region of the FTIR spectra of α-amylase in aqueous solutions of 0, 5, 10 and 20 mol% of EAN.

**Figure S4.** Amide I region of the FTIR spectra of β-lactoglobulin in aqueous solutions of 0, 5, 10 and 20 mol% of EAN.

**Figure S5.** Amide I region of the FTIR spectra of lysozyme in aqueous solutions of 0, 5, 10 and 20 mol% of EtAN.

**Figure S6.** Amide I region of the FTIR spectra of trypsin in aqueous solutions of 0, 5, 10, 20 and 30 mol% of EtAN.

**Figure S7.** Amide I region of the FTIR spectra of β-lactoglobulin in aqueous solutions of 0, 5 and 10 mol% of EtAN.

**Figure S8.** Amide I region of the FTIR spectra of α-amylase in aqueous solutions of 0 and 5 mol% of EtAN.

**Figure S9.** Amide I region of the FTIR spectra of lysozyme in aqueous solutions of 0, 5, 10, 20, 30 and 50 mol% of EAF.

**Figure S10.** Amide I region of the FTIR spectra of trypsin in aqueous solutions of 0, 5, 10, 20, 30 and 50 mol% of EAF.

**Figure S11.** Amide I region of the FTIR spectra of lysozyme in aqueous solutions of 0, 5, 10, 20, 30 and 50 mol% of EtAF.

**Figure S12.** Amide I region of the FTIR spectra of trypsin in aqueous solutions of 0, 5, 10, 20, 30 and 50 mol% of EtAF.

**Figure S13.** Amide I region of the FTIR spectra of β-lactoglobulin in aqueous solutions of 0, 5, 10, 20 and 30 mol% of EtAF.

**Figure S14.** Amide I region of the FTIR spectra of lysozyme in aqueous solutions of 0, 5, 10, 20, 30 and 50 mol% of EAG.

**Figure S15.** Amide I region of the FTIR spectra of trypsin in aqueous solutions of 0, 5, 10, 20 and 30 mol% of EAG.

**Figure S16.** Amide I region of the FTIR spectra of α-amylase in aqueous solutions of 0, 5, 10, 20 and 30 mol% of EAG.

**Figure S17.** Amide I region of the FTIR spectra of β-lactoglobulin in aqueous solutions of 0, 5, 10, 20 and 30 mol% of EAG.

**Figure S18.** Amide I region of the FTIR spectra of lysozyme in aqueous solutions of 0, 5, 10, 20 and 30 mol% of EtAG.

**Figure S19.** Amide I region of the FTIR spectra of trypsin in aqueous solutions of 0, 5 and 10 mol% of EtAG.

**Figure S20.** Amide I region of the FTIR spectra of α-amylase in aqueous solutions of 0 and 5 mol% of EtAG.

**Figure S21.** Amide I region of the FTIR spectra of β-lactoglobulin in aqueous solutions of 0, 5, 10, 20 and 30 mol% of EtAG.

**Figure S22.** Amide I region of the FTIR spectra of α-amylase in aqueous solutions of 0, 5, 10 and 20 mol% of TEtAA.

**Figure S23.** Amide I region of the FTIR spectra of β-lactoglobulin in aqueous solutions of 0, 5, 10 and 20 mol% of TEtAA.

**Figure S24.** Amide I region of the FTIR spectra of lysozyme in aqueous solutions of 0, 5, 10 and 20 mol% of TEtAA.

**Figure S25.** Amide I region of the FTIR spectra of trypsin in aqueous solutions of 0, 5, 10, 20, 30 and 50 mol% of TEtAA.

**Figure S26.** Amide 1 region of the FTIR spectra of Lysozyme in an aqueous solution of 10 mol% EAN. Spectra shown for measurements 1, 2 and 3 hours after synthesis on three separate samples.

1. Greaves, T. L.; Weerawardena, A.; Fong, C.; Krodkiewska, I.; Drummond, C. J., Protic Ionic Liquids: Solvents with Tunable Phase Behavior and Physicochemical Properties. *J. Phys. Chem. B* **2006,** *110* (45), 22479-22487.

2. Zhao, C.; Burrell, G.; Torriero, A. A. J.; Separovic, F.; Dunlop, N. F.; MacFarlane, D. R.; Bond, A. M., Electrochemistry of Room Temperature Protic Ionic Liquids. *J. Phys. Chem. B* **2008,** *112* (23), 6923-6936.

3. Burrell, G. L.; Burgar, I. M.; Separovic, F.; Dunlop, N. F., Preparation of Protic Ionic Liquids with Minimal Water Content and N-15 NMR Study of Proton Transfer. *Phys. Chem. Chem. Phys.* **2010,** *12* (7), 1571-1577.

4. Pinkert, A.; Ang, K. L.; Marsh, K. N.; Pang, S., Density, Viscosity and Electrical Conductivity of Protic Alkanolammonium Ionic Liquids. *Phys. Chem. Chem. Phys.* **2011,** *13*, 5136-5143.
